# Perioperative Management of Incidental Pulmonary Embolisms on Trauma CT Scans: A Narrative Review

**DOI:** 10.7759/cureus.34469

**Published:** 2023-01-31

**Authors:** Essam I Rama, James F Adeosun, Azeem Thahir, Matija Krkovic

**Affiliations:** 1 School of Clinical Medicine, University of Cambridge, Cambridge, GBR; 2 Trauma and Orthopaedic Surgery, Addenbrooke's Hospital, Cambridge University Hospitals NHS Foundation Trust, Cambridge, GBR

**Keywords:** trauma, computed tomography, inferior vena cava filters, anticoagulation, thromboprophylaxis

## Abstract

Unsuspected pulmonary embolism (PE) may be identified on an initial trauma computed tomography (CT) scan. The clinical importance of these incidental PEs remains to be elucidated. In patients who require surgery, careful management is needed. We sought to investigate the optimal perioperative management of such patients, including the use of pharmacological and mechanical thromboprophylaxis, possible thrombolytic therapy, and inferior vena cava (IVC) filters.

A literature search was conducted, and all relevant articles were identified, investigated, and included. Medical guidelines were also consulted where appropriate. Pharmacological thromboprophylaxis is the mainstay of preoperative treatment, and low-molecular-weight heparins, fondaparinux, and unfractionated heparin may all be used. It has been suggested that prophylaxis should be administered as soon as possible after trauma. Such agents may be contraindicated in patients with significant bleeding, and mechanical prophylaxis and inferior vena cava filters may be favoured in these patients. Therapeutic anticoagulation and thrombolytic therapies may be considered but are associated with an increased risk of haemorrhage. Delaying surgery might help to minimise the risk of recurrent venous thromboembolism, and any interruption of prophylaxis must be strategically planned. Recommendations for postoperative care include a continuation of prophylaxis and therapeutic anticoagulation, with follow-up clinical evaluation within six months.

Incidental PE is a common finding on trauma CT scans. Although the clinical significance is unknown, careful management of the balance between anticoagulation and bleeding is needed, especially in trauma patients and even more so in trauma patients requiring surgery.

## Introduction and background

A pulmonary embolism (PE) is a partial or complete occlusion of a branch of the pulmonary artery [1]. PE is a frequent complication of trauma injuries [2] and may be identified on an initial contrast-enhanced computed tomography (CT) scan.

The reported incidence of trauma-associated PE varies drastically between studies; Netto et al. [3] found an incidence of 2.2% across 1259 blunt trauma patients, which parallels the 2.3% found by O’Malley and Ross [4] in a similar-sized patient cohort. In a recent UK study, the incidence was exactly doubled [5], and where contrast-enhanced CT was performed irrespective of symptoms, an incidence of 24% was found [6]. It is unclear what exactly accounts for this wide range of reported incidences, but Shuster et al. [7] suggested that a large amount of the variability may be attributed to population characteristics and screening protocols.

Further questions remain regarding the temporal and etiological characteristics of PE after trauma injury. The view that PE may arise as a result of peripheral venous thromboembolism (VTE) that migrates to the lungs is in line with the notion of immobilisation having a significant causative role and therefore matches the traditionally held view that most PEs occur within the first five to seven days of injury. However, Owings et al. [8] suggested that up to 25% of PE may occur within four days, and there have been numerous reports of PEs occurring both within days [4,5,9] and hours of traumatic injury [10,11]. The prevalence of such rapidly identified incidental PEs has led to their temporal classification as immediate, early, or late, but the exact parameters differ between groups [3,5]. It has been suggested that this temporal distinction is not arbitrary but represents a difference in the underlying pathophysiology of early and late PEs associated with trauma [12], where immediately diagnosed PEs may be formed de novo as a result of a localised hypercoagulable state induced by trauma. This is further supported by the unlikelihood that a VTE would form and embolise within hours of injury. These are important considerations as they will have implications for clinical treatment and management of the patient. This article will discuss the perioperative management of PEs present on an initial trauma CT scan.

## Review

Methods

A literature search was conducted using PubMed, Medline, and Embase. Key search terms included: “pulmonary embolism,” “injury,” and “trauma,” and all the relevant publications were identified, with non-peer-reviewed items being excluded. Papers were not excluded based on the date of publication or language if a translation was available. Papers were initially selected according to the relevance of their titles, followed by the screening of abstracts and, if deemed to be suitable, the full text. Additional references cited by a paper were also investigated and included as necessary.

Discussion

Preoperative Management

Diagnosis of PE: The most common symptoms of PE are tachypnoea and dyspnoea; however, the symptoms are nonspecific, and incidental PEs are frequently asymptomatic [3]. In trauma patients, diagnosis is further complicated by frequent intubation, possible coma, or adequate explanations of symptoms by associated injury. Conventional diagnosis of PE is a multistep process. Patients who are suspected of having PE are initially evaluated to ascertain their acute risk of mortality due to PE according to their hemodynamic stability. Patients who are hemodynamically unstable (e.g., those with cardiogenic shock or arterial hypotension) are considered to have high-risk PE, and patients who are hemodynamically stable are considered to have non-high-risk PE [1]. This stratification informs the subsequent diagnostic procedures and treatment protocols, which allows them to be flexible in situations where urgency is required. Patients with high-risk (acute life-threatening) PE will undergo multidetector CT where imaging of the pulmonary arteries can confirm or exclude the diagnosis. Validated clinical decision rules such as the Wells [13] or Le Gal [14] rules can be used to determine the pretest probability of PE in patients with non-high-risk PE [15,16]. Patients in whom PE has not been ruled out should undergo D-dimer [17] and possible cardiac biomarker measurements [18,19] for further confirmation, while those with a high suspected clinical probability of PE should undergo imaging studies.

Imaging: With the introduction of high-resolution multidetector-row computed tomography (MDCT), CT pulmonary angiography (CTPA) has replaced catheter pulmonary angiography and ventilation-perfusion scintigraphy as the gold standard diagnostic imaging procedure for suspected PE [20,21]. The need for extensive screening to choose the appropriate diagnostic protocols is circumvented in instances of trauma by the common use of contrast-enhanced CT scans as part of the initial trauma assessment. For example, Netto et al. [3] reported that 64% of their patient cohort received a CT scan as part of their initial assessment. CTPA allows direct visualisation of the pulmonary arteries, at least to the segmental level [22], and CT-assessed right ventricular function adds further information regarding hemodynamic stability and hence provides prognostic value [23,24]. Although the Prospective Investigation of Pulmonary Embolism Diagnosis II (PIOPED II) study has described a high sensitivity and specificity of CTPA [25], this does not rule out the false-positive filling defects. Therefore, transthoracic echocardiography can also be used to confirm the diagnosis or as an alternative in cases where CT is not possible. Transthoracic echocardiography can also be used to measure right ventricular function. Further use of bilateral Doppler ultrasound of the limb veins or CT venography of the deep leg veins is then needed to confirm or exclude DVT and to inform the pathway for subsequent treatment [26,27]. In addition, novel artificial intelligence algorithms, such as Aidoc, may become increasingly useful in assisting diagnosis due to their high specificity and sensitivity in the detection of incidental PE [28].

Treatment of PE: Treatment of trauma associated with PE varies between trauma centres. Thromboprophylaxis is the mainstay of treatment, and therapeutic anticoagulation may be used if DVT is present. Mechanical means of thromboprophylaxis may also be used. Physical interventions such as inferior vena cava (IVC) filters, as well as the use of pharmacological thrombolytics or pulmonary embolectomy, may be considered. Patients requiring surgery are also at an increased risk of thromboembolic events, so anticoagulant treatment should also be influenced by surgical considerations. Further complication is added by the risk of bleeding in trauma patients, which is exacerbated by anticoagulant use.

The use of thromboprophylaxis in trauma patients is complicated by the need to balance the risk between thromboembolic events and possible haemorrhage; historically, this has resulted in delayed prophylaxis administration. Indeed in individuals with significant bleeding and/or intracranial haemorrhage, prophylaxis may be avoided completely [10]. However, Owings et al. [8] have shown that patients who did not receive prophylaxis had an earlier PE diagnosis, and it has been suggested that prophylaxis should be administered as soon as possible after trauma injury, i.e., when the risk of bleeding is suitably low. The wide variation in trauma injury presentation means that prophylactic treatment may require tailoring on an individual case-by-case basis and that general guidelines for PE treatment may not be directly applicable to those trauma patients [7].

Unfractionated heparin (UFH), low-molecular-weight heparin (LMWH), and the synthetic fondaparinux are the cornerstones of pharmacological prophylaxis. For trauma patients undergoing surgery, thromboprophylaxis serves a dual function, and the surgery as well as patient contraindications will influence the treatment protocol. LMWH is suitable for general and orthopaedic surgery, whereas fondaparinux may be preferred in abdominal, thoracic, or cardiac surgery as well as in patients with lower limb immobilisation or fragility fractures of the hip or femur [29]. Due to their renal elimination, LMWHs and fondaparinux should be avoided in patients with renal impairment, in favour of UFH. A typical dose of LMWH may be 5000 units administered subcutaneously once daily, or twice daily when there is a particularly high risk of thromboembolic events [3].

LMWHs, fondaparinux, and UFH may also be used as therapeutic anticoagulation where proximal DVT is present. The direct inhibitors of factor Xa, dabigatran, apixaban, and rivaroxaban are also used and have shown comparable effectiveness to warfarin [30]. LMWH should be combined with the administration of vitamin K antagonists (VKAs) from the first or second day, and LMWH or fondaparinux treatment is continued for at least five days or until the international normalised ratio (INR) is between 2 and 3 on two consecutive days [1,29]. Aside from restoring pulmonary blood flow, therapeutic anticoagulation aims to prevent recurrent thromboembolic events, and the current recommendations suggest continuing anticoagulant therapy for three months in cases where the event was secondary to a removable risk factor [21].

An interesting question is whether incidental PE in trauma patients actually requires treatment. It might be posited that the risk of fatal or recurrent PE as a result of immediate PE necessitates early treatment, but arguments can be made against incidental PE treatment in trauma patients. Indeed, the clinical significance of incidental PE and thus the need for therapeutic anticoagulation has been questioned [3]. Schultz et al. showed that no recurrent VTE was found in 17 trauma patients with incidental PE who did not receive therapeutic anticoagulation [6]. Further, anticoagulation is associated with complications such as gastrointestinal (GI) bleeding and haemothorax reaccumulation [31], and anticoagulation preceding surgery is associated with increased minor and major bleeding during surgery and surgical site bleeding post-surgery [32]. It is clear that further refinement of the criteria necessitating therapeutic anticoagulation in incidental PE trauma patients is needed.

Mechanical prophylaxis includes anti-embolism stockings (also called thromboembolism deterrent stockings [TEDs]) and intermittent pneumatic compression (IPC) devices. Stockings provide a graduated compression of the calf, and IPC devices use air-filled cuffs that squeeze the lower limbs. While mechanical prophylaxis tends to be less efficacious than the pharmacological counterpart, the incidence of VTE has been shown to be reduced by up to 60% when compared with no treatment [33]. Mechanical prophylaxis is commonly offered to patients with major trauma and those undergoing various elective surgeries and is particularly important when pharmacological prophylaxis is contraindicated due to the risk of haemorrhage, e.g., in neurosurgical patients [29].

Acute right heart failure is the main cause of death in PE patients with hemodynamic instability [1]. Therefore, immediate thrombolytic treatment is needed for hemodynamically unstable patients with confirmed PE. Alteplase is commonly used either as an IV bolus followed by constant infusion or IV infusion over 2 hours, as are urokinase and streptokinase in similar protocols. Thrombolytic therapy may be contraindicated in trauma patients due to the increased risk of major bleeding and haemorrhagic stroke associated with it. As a result, alternative interventional treatments such as pulmonary catheter thrombolysis, thrombus aspiration, and catheter or surgical thrombectomy may be considered. Catheter thrombolysis is appropriate for patients with submassive PE, whereas surgical thrombectomy is better suited to patients with massive PE [20]. The possibility of embolectomy will depend on the accessibility of the pulmonary embolus within the arterial circulation, for example, distally located emboli may not be treatable by this method [10]. Patients presenting with incidental PEs are often asymptomatic; Netto et al. [3] showed that all six patients with immediate PE were asymptomatic (hemodynamically stable). Therefore, the utility of thrombolytic therapy in incidental PE in trauma patients would appear to be low.

IVC filters serve the function of trapping thromboemboli from the deep veins of the lower limb before they enter the pulmonary circulation [34]. Although the systematic use of IVC filters for preventing recurrent PE is not recommended [35], they may be used when therapeutic anticoagulation is contraindicated, when anticoagulation has failed to prevent PE, or in patients with prolonged immobilisation [1,8,10,34]. Although IVC filter usage is controversial, there is evidence that they do not reduce the incidence of clinically important VTE [36,37]. Initiation of traditional anticoagulant treatment is recommended when the risk of bleeding resolves [35].

Management of potential bleeding: Trauma injuries may often be associated with bleeding. Bleeding can be classified as major or minor, where major bleeding is associated with hemodynamic instability [38]. Careful management of potential bleeding is required in trauma patients with PE who have been treated with anticoagulants. If major bleeding is confirmed, chemoprophylaxis should be stopped, and blood transfusion or reversal agents may be needed. Discontinuation of treatment is not necessary for minor bleeding. The timeframe for restarting anticoagulation after bleeding will vary according to the site of bleeding and, as with the initial chemoprophylactic administration, will vary on a case-by-case basis.

Surgery for Traumatic Injury

The surgical requirements after traumatic injury depend on the extent of the injury, and the variety of clinical presentations makes formulating treatment algorithms difficult. The patient's condition may range from clinically stable to “in extremis,” and this will influence the optimal route for surgical treatment. For example, patients may undergo emergency life-saving damage control surgery (DCS) or definitive fixation of all long bone fractures within 24 hours with early total care (ETC) [39-41].

Considerations for the timing of prophylaxis: In cases where surgery is performed shortly (<24 hours) after trauma and/or where significant bleeding is involved, it may not be possible to administer anticoagulant medication until days after the surgery when the patient is stable and the risk of haemorrhage is lower [11]. However, it has been shown that in hip fracture patients, there was no significant difference in the incidence of intraoperative and postoperative bleeding between preoperatively and postoperatively initiated LMWH therapy, in favour of preoperative prophylaxis where possible [42]. Another possibility is delaying surgery. O’Donnell and Kearon have recommended postponement of elective surgery for a minimum of two to four weeks from the acute PE event due to the high risk of recurrent VTE within the first month [43], but delaying surgery may not be an option for trauma patients, and the use of IVC filters is recommended in these cases. Furthermore, delaying surgery may not be necessary; Kim et al. have presented evidence in support of early orthopaedic surgery as a treatment option in patients with acute PE [44]. In instances where surgery is delayed, careful consideration of anticoagulant interruption (in order to minimise perioperative bleeding) is required. The importance of continuous prophylaxis cannot be understated; interruption of VTE prophylaxis for even a day is related to an increased risk of VTE, so preoperative withdrawal requires strategic planning [45]. In patients with PE, VKA interruption may occur five days before surgery, but the high risk of recurrent VTE means that bridging therapy may often be initiated, e.g., administration of subcutaneous LMWH for 10 to 12 days during VKA interruption [46]. Overall, the balance between the patient's risk for VTE and perioperative bleeding is the primary concern in the perioperative management of anticoagulant therapy.

Intraoperative Management

Patients undergoing surgery with incidental PE require close monitoring of hemodynamic stability. Sudden falls in pulmonary end-tidal CO_2_ and increased pulmonary arterial CO_2_ may be indicative of the effects of PE [47]. For patients without IVC filters already fitted, these may be considered. The possible use of thrombolytic or anticoagulant therapy must be weighed against the risk of bleeding as they would be preoperative. Surgery is also a contraindication for thrombolysis.

Postoperative Management

Postoperative anticoagulation: In 2004, Pengo et al. found the rate of recurrent VTE to be around 8% within one year of the initial embolic event [48], and Schulman et al. [49] found that the incidence of recurrence was higher in patients with prior PE compared to those with DVT. The optimal postoperative dose of pharmacological prophylaxis is unknown. In patients undergoing major orthopaedic surgery, prophylaxis may continue up to 35 days from the date of surgery [50], and patients undergoing other major surgeries or those who have spinal injuries may also be considered for post-discharge prophylaxis. Indeed, when compared to thromboprophylaxis during the hospital stay only, prolonged pharmacological prophylaxis reduces the risk of recurrent VTE and does not increase bleeding or mortality after major surgery [51]. Therapeutic anticoagulation is recommended for at least three months in all patients with PE, and discontinuation of oral anticoagulation is recommended after three months in all patients with PE secondary to trauma [52].

Post-PE care and long-term complications: Around 80% of patients with acute PE will survive the initial event. However, chronic vascular changes following emboli put patients at risk of two sequelae: chronic thromboembolic pulmonary hypertension (CTEPH) and post-thrombotic syndrome [53]. Routine clinical evaluation is recommended between the first three and six months of acute PE. After three months of acute PE, symptomatic patients with mismatched perfusion defects on ventilation/perfusion (V/Q) scans should be referred to a CTEPH centre for further treatment.

Removal of IVC filters: While some authors suggest the removal of IVC filters as soon as possible to prevent secondary vena cava thromboses [1], others have suggested that extended retrieval intervals are both safe and may serve to maximise protection against recurrent PE [54].

## Conclusions

Incidental PE is a common finding on trauma CT scans. Questions remain regarding the etiological and temporal factors involved such as the relative importance of localised hypercoagulability and inflammation compared to the possibility of DVT emboli arising from the periphery. LMWH and fondaparinux are the mainstays of pharmacological thromboprophylaxis, but the use of such agents must be balanced against the risk of bleeding. It is beneficial to start prophylactic treatment as soon as possible, except in patients with major trauma or those undergoing cranial surgery due to the significant risk of haemorrhage. Mechanical prophylaxis, such as anti-embolism stockings or IPC, is the first-line treatment in these patients. The need for therapeutic anticoagulation, as well as the efficacy of IVC filters, is unclear. Surgery for traumatic injury cannot usually be postponed, but elective procedures should be postponed for at least three months until completion of the course of a direct-acting oral anticoagulant (e.g., apixaban or rivaroxaban). Pharmacological prophylaxis should be continued after surgery and may be extended for up to five weeks following major orthopaedic surgery. Therapeutic anticoagulation is recommended for a minimum of three months and may be continued for a further three months in unprovoked PE. Follow-up evaluation and monitoring for clinical sequelae should occur within three to six months.
